# Metabolomic Profiling and Molecular Networking of Nudibranch-Associated *Streptomyces* sp. SCSIO 001680

**DOI:** 10.3390/molecules27144542

**Published:** 2022-07-16

**Authors:** Samar M. Abdelrahman, Noura S. Dosoky, Amro M. Hanora, Nicole B. Lopanik

**Affiliations:** 1 School of Earth and Atmospheric Sciences, Georgia Institute of Technology, Atlanta, GA 30332, USA; nicole.lopanik@cancer.org; 2 Department of Botany and Microbiology, Faculty of Science, Suez University, Suez 43518, Egypt; 3 Aromatic Plant Research Center, Lehi, UT 84043, USA; ndosoky@aromaticplant.org; 4 Department of Microbiology and Immunology, Faculty of Pharmacy, Suez Canal University, Ismailia 41522, Egypt; ahanora@yahoo.com; 5 School of Biological Sciences, Georgia Institute of Technology, Atlanta, GA 30332, USA; 6 American Cancer Society, Atlanta, GA 30303, USA

**Keywords:** natural products, associated microbiomes, metabolites, *Streptomyces*, high-resolution mass spectrometry, Global Products Social Molecular Networking

## Abstract

Antibiotic-resistant bacteria are the primary source of one of the growing public health problems that requires global attention, indicating an urgent need for new antibiotics. Marine ecosystems are characterized by high biodiversity and are considered one of the essential sources of bioactive chemical compounds. Bacterial associates of marine invertebrates are commonly a source of active medicinal and natural products and are important sources for drug discovery. Hence, marine invertebrate-associated microbiomes are a fruitful resource for excavating novel genes and bioactive compounds. In a previous study, we isolated *Streptomyces* sp. SCSIO 001680, coded as strain 63, from the Red Sea nudibranch *Chromodoris quadricolor*, which exhibited antimicrobial and antitumor activity. In addition, this isolate harbors several natural product biosynthetic gene clusters, suggesting it has the potential to produce bioactive natural products. The present study aimed to investigate the metabolic profile of the isolated *Streptomyces* sp. SCSIO 001680 (strain 63) and to predict their potential role in the host’s survival. The crude metabolic extracts of strain 63 cultivated in two different media were characterized by ultra-high-performance liquid chromatography and high-resolution mass spectrometry. The metabolomics approach provided us with characteristic chemical fingerprints of the cellular processes and the relative abundance of specific compounds. The Global Products Social Molecular Networking database was used to identify the metabolites. While 434 metabolites were detected in the extracts, only a few compounds were identified based on the standards and the public spectral libraries, including desferrioxamines, marineosin A, and bisucaberin, halichoblelide, alternarin A, pachastrelloside A, streptodepsipeptide P1 1B, didemnaketal F, and alexandrolide. This finding suggests that this strain harbors several novel compounds. In addition, the metabolism of the microbiome of marine invertebrates remains poorly represented. Thus, our data constitute a valuable complement to the study of metabolism in the host microbiome.

## 1. Introduction

Natural products (NPs) have played a key role in drug discovery, especially in cancer and infectious diseases. Hence, screening for new bioactive natural compounds is critical to detect new bioactive compounds with therapeutic effects that can help fight debutant diseases, stop the spread of infectious agents, and contest the drug-resistant bacteria that rise to highly dangerous levels and harbor new resistance mechanisms and that threaten our ability to treat common, and sometimes incurable, infectious diseases as antibiotics become less effective. Natural products represent 42% of the total approved drugs from 1981 to 2019 and are the source for 56% of approved antibacterial drugs [1]. Many bioactive compounds have been isolated in the marine environment due to their high diversity in invertebrates, including sponges and Mollusca [2]. Such marine invertebrates are frequently colonized by various microorganisms, which can provide multiple benefits to their host organism [3]. For example, invertebrates do not possess antibody-based immunity but can eliminate pathogens by building an adaptive immune system via associated microbiomes [4]. *Hydra* is a genus of freshwater invertebrates that mount a robust immune response from their associated microbiome, even without motile phagocytic cells. This proposes that the microbiome plays a crucial role in *Hydra*’s innate immune system [4,5].

Microbial symbionts often interact with host cells through the production and degradation of metabolic compounds [6]. A study of metabolic cooperation in *Cymbastela concentrica* sponge and two of its bacterial symbionts presented that the creatine and creatinine produced by the sponge’s metabolism are decayed into glycine by its symbionts [7]. In another example, comparative metabolomics analysis of extracts from the sponge *Haliclona simulans* and one of its microbial associates (the actinomycete *Streptomyces* sp.) demonstrated the presence of common metabolites in both extracts [8]. It can be concluded that the microbiome is a critical producer of peculiar bioactive compounds that play crucial roles in the host’s adaptation to environmental conditions [4] and offer a robust resource for drug discovery.

Ala-geninthiocin, a new thiopeptide antibiotic with potent cytotoxicity against A549 human lung carcinoma, was recently identified from marine *Streptomyces* sp. ICN19 [9]. Therefore, microbial communities associated with marine invertebrates have gained considerable attention. A large number of bacterial species are associated with marine invertebrates, such as Actinobacteria, Bacteroidetes, Acidobacteria, Planctomycetes, and Chloroflexi [10,11]. Actinobacteria are Gram-positive filamentous bacteria that possess many biosynthetic gene clusters. Roughly 10,000 bioactive metabolites have been isolated from Actinobacteria, which comprise 45% of the total bioactive microbial metabolites found [12,13,14]. *Streptomyces* produces secondary metabolite compounds such as siderophores that help sequester iron, pigments that protect them from UV radiation, and antibiotics that inhibit competitors and facilitate communication with other species. The discovery of streptomycin from *Streptomyces* has inspired the research of antibiotics from the genus *Streptomyces*, which is one of the predominant sources of new bioactive molecules, representing 62.5% of the total bacterial marine natural products (MNPs) that have been reported recently [15,16]. *Streptomyces* bioactive molecules are used in clinical, industrial, and pharmaceutical applications, including antibacterial, antifungal, antiviral, antihypertensive, immunosuppressive, and antitumor compounds [17,18,19,20]. Following the genomic sequencing of various *Streptomyces* species, 12 secondary metabolite gene clusters were found to exist in almost all species of *Streptomyces*. The genomic analysis of thirty *Streptomyces* strains resulted in the identification of a total of 922 clusters, but most of the compounds detected and encoded by these gene clusters are unknown [21,22,23]. Consequently, *Streptomyces* is a strong resource for natural products and an ideal organism for novel compound discovery.

Traditional biochemical screening is not well-suited for the discovery of novel drug candidates because it is resource-intensive, time-consuming, and allows for matrix interference [24]. In contrast, metabolomics provides a unique fingerprint for biological systems that can overcome several of the aforementioned problems using fast and accurate methods, primarily high-performance liquid chromatography combined with high-resolution tandem mass spectrometry (HPLC-MS/MS) [25]. A comprehensive analysis of a wide range of metabolites can provide a dynamic understanding of intracellular metabolic states under different conditions by comparing the MS/MS datasets with data uploaded on open-source repositories [26].

Metabolomic profiling provides a unique approach to pursuing traditionally understudied bacteria. The present study aimed to investigate the metabolic profile of the isolated *Streptomyces* sp. SCSIO 001680 (strain 63) and to predict its potential role in the host’s survival. Therefore, to leverage the strength of the metabolomics, we assessed the metabolites yielded by strain 63, which is associated with *C. quadricolour*, a dorid nudibranch gathered from the Red Sea, using ultra-high-performance liquid chromatography–high-resolution mass spectrometry (UHPLC-HRMS/MS). To obtain a comprehensive understanding of the whole metabolic profile, two different media were used for cultivation, and both the pellets and filtrates were analyzed. The Global Natural Products Social Molecular Networking Project (GNPS), an open-access small molecule-focused data curation and analysis infrastructure [27], was used to generate a molecular network and to identify the metabolites. The dereplication of metabolites was later performed through the Marinlit platform to identify additional molecules. The metabolic profile of the bacteria associated with *C. quadricolor* was previously unknown, which added to the novelty of this work.

## 2. Results and Discussion

**Figure 1:**
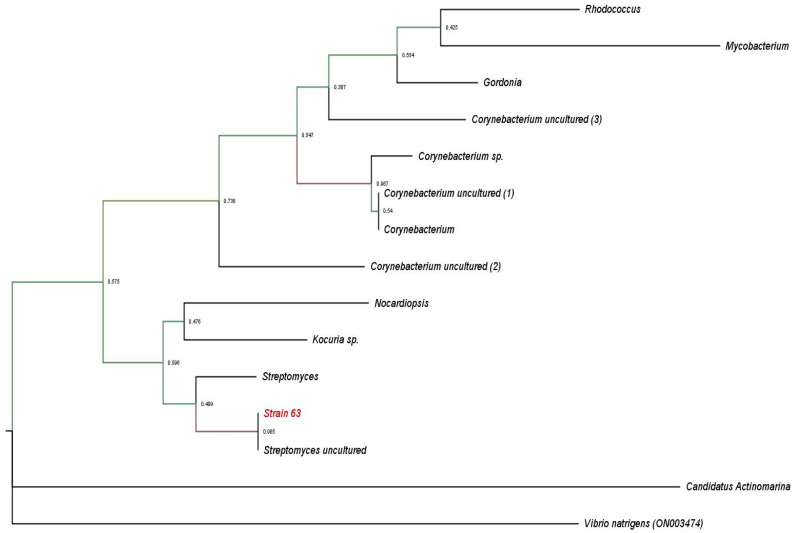
Molecularly rooted phylogenetic 16S rRNA gene tree analysis for strain 63 of *Streptomyces* sp. SCSIO 001680 by maximum likelihood method. The tree was rooted using 16S rRNA partial gene sequence of *Vibrio natrigens*- ON003437. The evolutionary history was inferred using the maximum likelihood method based on the Tamura3-parameter. Evolutionary analyses were conducted in MEGA 7 [41].

The marine environment is one of the major important sources of natural products, comprising a varied range of products of a unique ecological nature [28,29]. The bioprospecting of pharmaceuticals from marine environments increases the frequency of unique discoveries. The isolation of marine microorganisms, whether free in water or associated with marine invertebrates, are an important source of bioactive natural products [30,31,32]. Nudibranchs are a rich source of bioactive compounds but have mainly been studied from an ecological perspective, and there are few studies on the natural products of nudibranch-associated microbes [33,34]. There has only been one precocious study of the natural products of the dorid nudibranch *C. quadricolor*, which stated that anticancer latrunculins A and B were insulated from their diet [35]. Metabolites produced by microbial associates can contribute to the defense of nudibranchs [36], and Chormodorididae nudibranchs have diverse mechanisms for obtaining defensive chemicals for storage in tissues [37]. While many clinically important pharmaceuticals have already been discovered from the order Actinomycetales [14], the species associated with marine ecosystems are also rich sources of bioactive metabolites [38,39,40]. The 16S rRNA sequence of strain 63 was aligned with associated microbiome sequences that were previously generated for *C. quadricolor* to ensure the association of strain 63 with the animal. Sequence data suggest that the most dominant members of the nudibranch microbiome have not been captured by the culture. *Streptomyces* sp. represented 26% of the total Actinomycetes associated with *C. quadricolor*, which is a small fraction of all nudibranch skin microbiota. Strain 63 inhabits the animal skin tissue, with 100% similarity to one of the microbiome sequences (Figure 1). It is enticing to presume that *Streptomyces* sp. lives in a mutualistic association with *C. quadricolor* and protects them against bacterial infections.

### 2.1. Metabolic Profile and Molecular Networking Analysis

**Figure 2:**
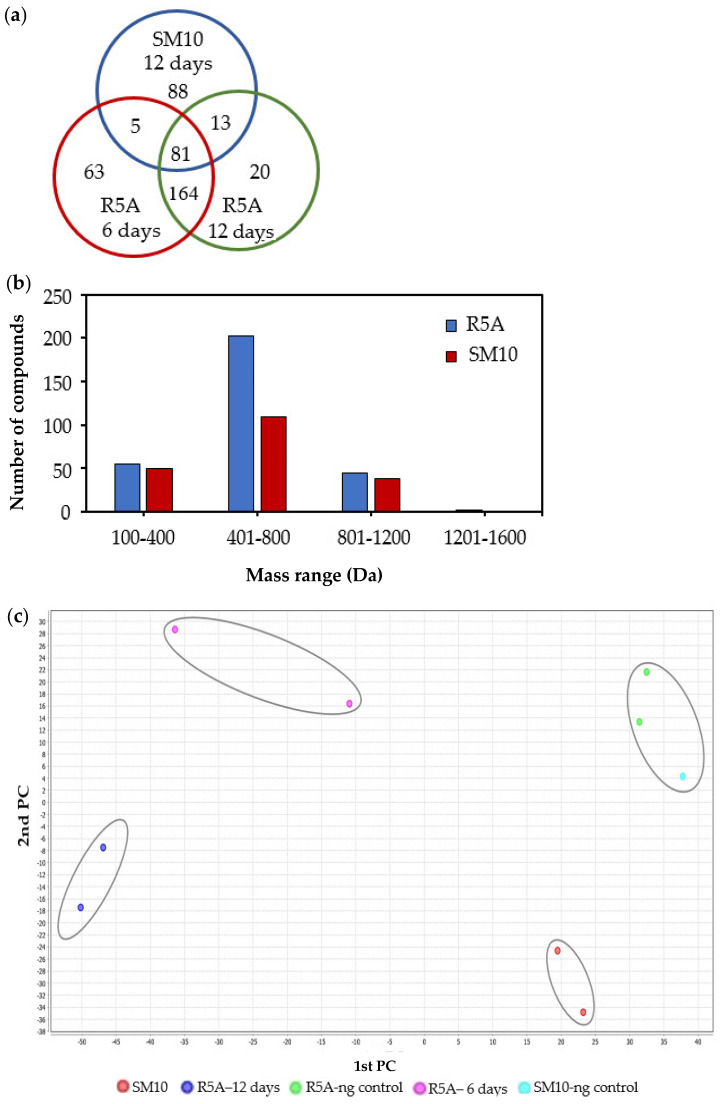
Metabolites of *Streptomyces* sp. SCSIO 001680: (**a**) unique and common metabolites after growth in R5A and SM10 media, (**b**) number of metabolites (masses) within mass ranges for each media, and (**c**) two-dimensional plot of metabolites produced in SM10 and R5A media by PCA analysis.

**Figure 3:**
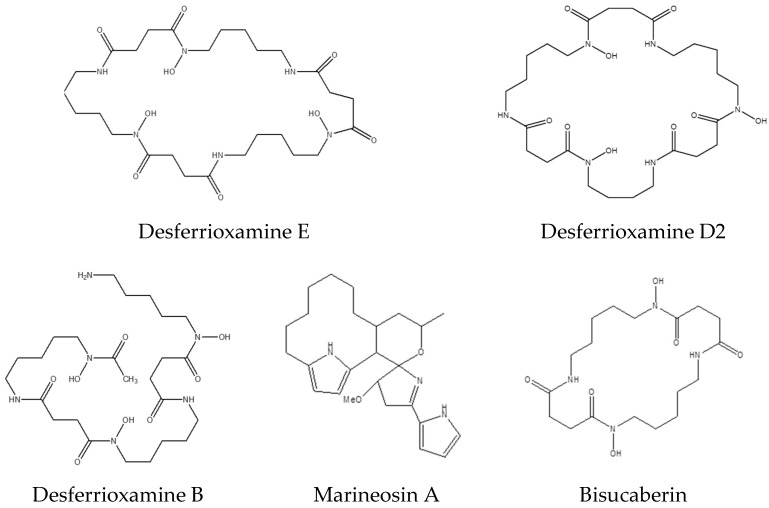
Identified compounds produced by *Streptomyces* sp. by GNPS presented by the node and the edge graph.

In the current study, the metabolic profile of strain 63 associated with *C. quadricolor* was examined by dereplicating LC-MS/MS+ for the combined culture pellet and supernatant extract in two culture media: SM10 and R5A, for 6 and 12 days. Differences in the LC-MS/MS + chromatograms of the culture extracts from the two media suggest that strain 63’s secondary metabolite production varies under the two conditions. The MS/MS data obtained from positive ion mode after charge correction, the elimination of adducts, and background and media subtraction were used to build a network using GNPS (Figure S1). The molecular network contained 859 nodes, and the molecular size of the parent mass ranged from 177 to 1403 Da. The metabolites varied considerably among the different media. Metabolites were annotated by matching spectra against public libraries, and a total of 11 compounds were detected and identified. A total of 434 metabolites with distinct parent masses and fragment spectra were detected, of which 81 metabolites were found to be common metabolites between the media. The increased number of bacterial metabolites produced through growth in the R5A medium compared to in the SM10 medium is likely due to the richer carbon sources in the composition of the R5A medium. Although fewer metabolites were produced in SM10 than in R5A, it had 88 distinctive metabolites (Figure 2a). A greater number of metabolites with a high molecular mass were detected in R5A than in SM10 (Figure 2b). Metabolites with a mass ranging from 1200 to 1600 Da were only found in R5A. Principal component analysis (PCA) plots of the metabolomics profiles were generated for 6 and 12 days in both media (Figure 2c). The PCA plot demonstrated distinctive patterns in the bacterial metabolites on different media, and the replicates of each media were distinctly grouped together. Distinct separation between the R5A and SM10 metabolomics profiles was observed, indicating that *Streptomyces* strain 63’s metabolism was different in one medium compared to the other. The overall metabolite profile demonstrated a diversity of natural products, including lipids, amines, cyclic peptides, steroids, and polyamines. The detected metabolites included desferrioxamines, marineosin A, and bisucaberin (Figure 3).

Desferrioxamines are potent multidentate iron binders, also known as siderophores [42], and have been previously characterized in *Streptomyces*. Clinically, desferrioxamines are used to treat iron overload and metal toxicity [43]. Desferrioxamine E (Nocardamine [M + Na]^+^, *m*/*z* 623.339), desferrioxamine B (Desferan [M + H]^+^, *m*/*z* 561.361), and desferrioxamine D2 (demethylenenocardamine, [M + H]^+^, *m*/*z* 587.342) were found in extracts from strain 63 grown in R5A medium. Desferrioxamine E is a dimeric cyclic peptide with antitumor activity against melanoma (skin cancer) [44]. Desferrioxamine B is used to treat metal ion dyshomeostasis [45] and radiometal-based imaging [46]. The desferrioxamine D2 that was isolated from *Streptomyces* nicoyae exhibited antimicrobial activity against Uropathogenic *E. coli* (UPEC) [47]. Bisucaberin, a dihydroxamate siderophore ([M + H]^+^, *m*/*z* 401.251), was also discovered in the *Streptomyces* extract in SM10 medium. Bisucaberin was previously isolated from *Alteromonas haloplanktis* strain SB-1123 and demonstrated direct cytotoxicity on tumor cells [48]. The ion corresponding to marineosin A ([M + H]^+^, *m*/*z* 410.291), which is a cytotoxic agent against colon tumor cell lines [49], was also detected in the *Streptomyces* extract in both R5A and SM10 media (Figure 3 and Table S1).

### 2.2. Marinlit Database Annotation

**Figure 4:**
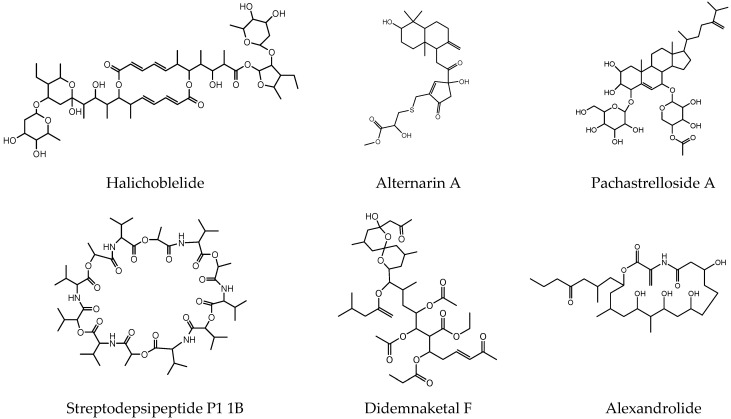
Dereplicated metabolites of *Streptomyces* sp. SCSIO 001680. These compounds were predicted from the Marinlit database.

Mining the Marinlit database for molecules detected by LC-MS/MS with high UV absorbance led to identifying additional molecules (Figure 4, Table S2). The ion peak at *m*/*z* 1039.578 [M + H]^+^ was dereplicated as the macrolide halichoblelide previously identified in *Streptomyces hygroscopicus* and that possessed cytotoxic activity [50]. The ion at *m*/*z* 1083.606 [M + H]^+^ was dereplicated as streptodepsipeptide P11B, a cyclodepsipeptide previously isolated from the marine *Streptomyces* sp. P11-23B, which proved to be cytotoxic against various glioma cell lines [51]. The mass ion peak at *m*/*z* 495.240 [M + H]^+^ was dereplicated as alternarin A, a drimane meroterpenoid that was recently characterized in coral-associated *Alternaria* sp. ZH-15 as a neuronal excitability suppressant [52]. The ion peak at *m*/*z* 528.352 [M + H]^+^ was identified as the diatom growth inhibitor alexandrolide, which was previously isolated from the dinoflagellate *Alexandrium catenella* [53]. The mass ion peak at *m*/*z* 783.453 [M + H]^+^ was dereplicated as pachastrelloside A, which was characterized in a marine sponge from the genus *Pachastrella* [54]. Finally, the mass ion peak at *m*/*z* 853.495 [M + H]^+^ was dereplicated as didemnaketal F, an antimicrobial metabolite reported from the ascidian *Didemnum* sp. with potent antimicrobial activity against *E. coli* and the fungus *Candida albicans.* Didemnaketal F also displayed moderate activity against HeLa cells [55]. It is noteworthy that the metabolites shown in (Figure 4), including alternarin A, alexandrolide, pachastrelloside A, and didemnaketal F, are reported here for the first time in the genus *Streptomyces*. 

Moreover, we studied the metabolomic profiling of *Streptomyces* associated with *C. quadricolor,* which possesses antibacterial and antitumor activity. Our analysis suggested that the *Streptomyces* metabolites varied when cultured with different media types. As expected, the metabolomic profile indicates that the culture grown in R5A possesses more diverse metabolites compared to that grown in SM10. For instance, all of the desferrioxamines, bisucaberin, α-bisabolol, and alternarin A were only detected from extracts of culture grown in R5A medium. In contrast, didemnaketal F was only detected from culture extracts grown in SM10 medium. In contrast, pachastrelloside A, marineosin A, alexandrolide, streptodepsipeptide P11B, and halichoblelide were detected in both media after the 12-day incubation periods. 

Many marine invertebrates, including sponges, bryozoans, and ascidians, have been shown to obtain metabolites from their microbial associates in a symbiotic relationship [56,57]. Previous studies report that nudibranchs utilize a diverse range of chemical defenses from different sources, including diet, de novo synthesis, and microbes [37]. The detection of many compounds with cytotoxic activity and antimicrobial activity suggests that *Streptomyces* strain 63 could contribute to the defense of *C. quadricolor* from fungi, bacterial infections, and predation. In our previous study of strain 63, the ethyl acetate extract of the liquid culture medium exhibited antimicrobial and antitumor activity, and genomic DNA was shown to harbor several biosynthetic gene clusters, including polyketide synthase (PKS; type I and type II) and non-ribosomal peptide synthetase (NRPS) gene clusters [58]. Our results reinforce the hypothesis that the nudibranch-associated microbiome is a significant source of bioactive compounds, which may support the nudibranch’s chemical defense. Moreover, based on our findings, the present approach indicates the utility of LC-MS/MS-based metabolomics to understand the natural product production of understudied marine bacteria associated with *C. quadricolor*. Finally, the associated microbiome is a strong contender for future genomic studies targeting biosynthetic gene clusters.

## 3. Materials and Methods

### 3.1. Investigate the Association of Strain 63 within C. quadricolor Microbiome

The sequences from several individual SVs classified as Actinomycetes within the *C. quadricolor* microbiome were aligned with the complete 16S rRNA gene sequence for strain 63 using Geneious Prime followed by BLASTn searches against the NCBI nr database. The phylogenetic tree was generated by the maximum likelihood method using Mega 7 [41] and visualized by FigTree (http://tree.bio.ed.ac.uk/software/figtree/ (accessed on 1 April 2022)). 

### 3.2. Streptomyces Cultivation

*Streptomyces* sp. SCSIO 001680 was preserved as a lyophilized sample. It was cultivated as previously described [58]. Briefly, strain 63 was inoculated on an isolation medium R2A plate and incubated at 30 °C for five days. A single colony was transferred into 5 mL of R2A liquid medium and incubated for 5 days in a rotary shaker (Thermo Scientific (Waltham, MA, USA), MaxQ 6000) at 150 rpm and 30 °C as a starter culture. The medium was prepared using filtered Instant Ocean Sea Salt (Instant Ocean, Blacksburg, VA, USA) with a salinity of 35 ppt.

### 3.3. Metabolite Extraction Using SPE Column

R5A and SM10 media were used to detect metabolites. Both media were prepared with filtered Instant Ocean Sea Salt (20 ppt salinity). The pH was adjusted to 6.85 and 6.5 for R5A and SM10, respectively, before autoclaving. Furthermore, 500 μL of the starter culture was used to inoculate two replicates of Erlenmeyer flasks containing 50 mL of medium and incubated at 30 °C for 12 days with shaking at 150 rpm. Amounts of 25 mL of the bacterial culture and the negative control were collected after 6 and 12 days for the R5A medium and after 12 days for the SM10 medium (as there was little growth in the first 6 days) to reveal the production profile of secondary metabolites. The cultures were centrifuged at 3500 rpm for 30 min. In order to detect a high number of secondary metabolites, both filtrate and cell pellet extraction were used. The filtrate metabolites were extracted by solid-phase extraction (SPE) using a 100 mg/mL Strata^®^ C18-E cartridge (55 µm, 70 A) (Phenomenex, Torrance, CA, USA). Using a positive pressure manifold, the organic compounds adsorbed on the C18 cartridge were eluted using 1 mL of water with 20%, 50%, 80%, and 100% acetonitrile. For cell pellet extraction, the pellet was frozen for 1 h at −80 °C. Subsequently, 5 mL of methanol was added to the cells, sonicated for 20 min, and centrifuged at 3500 rpm for 10 min. The methanolic extract of the pellet was added to the filtrate extract, dried by speed vacuum, and directly analyzed by LC-MS/MS.

### 3.4. UHPLC HRMS Analysis

An Agilent 1290 Infinity II UHPLC System (Santa Clara, CA, USA) combined with a Bruker ImpactII ultra-high-resolution Qq-TOF mass spectrometer (Bruker Daltonics, GmbH, Bremen, Germany) equipped with an electron spray ionization (ESI) source was used for metabolite analysis. A Kinetex™ 1.7 µm UHPLC (C18) column (50 × 2.1 mm) was used for chromatographic separation. MS spectra were acquired in positive ionization mode from *m*/*z* 50–2000 Da. Metabolic extracts of the *Streptomyces* strain were resuspended in 1 mL methanol (LC-MS grade) and directly analyzed. After injection with 10 µL of the metabolic extract, it was separated using a gradient of water (A) and acetonitrile 100% (B) with 0.1% formic acid and at a flow rate of 0.5 mL/min throughout the run. The gradient elution was initiated at 5% solvent B for 3 min and then at a linear gradient of 5% to 50% B over 5 min and held at 50% B for 2 min followed by a linear gradient of 50% to 100% B over 5 min and held at 100% B for 3 min. The column was then re-equilibrated to 5% B for 1 min.

### 3.5. Molecular Networking and Spectra Annotation

Mass spectrometry data were dereplicated using Global Natural Product Social Molecular Networking (GNPS) after converting the data files to mzXML format using MS–Convert software. The molecular network was generated from the positive ion mode using the online workflow (https://ccmsucsd.github.io/GNPSDocumentation/ (accessed on 1 March 2022)) on the GNPS website (http://gnps.ucsd.edu (accessed on 1 March 2022)) [27]. The created molecular network was visualized via Cytoscape 3.7.2. [59]. Annotation was first obtained by matching spectra in public libraries. To determine the clustering of metabolites within different media, principal component analysis (PCA) was generated using MZmine 2.53. [60]. The mass spectrometry data collected were deposited in the MassIVE repository with the MassIVE ID# MSV000089210.

## 4. Conclusions

Actinobacteria are crucial to drug discovery, especially the genus *Streptomyces*, which harbors many habitats. In several cases, *Streptomyces* sp. associates with other organisms to protect their host from bacterial infections. In this study, the metabolic extracts of *Streptomyces* sp. associated with *C. quadricolor* were characterized by ultra-high-performance liquid chromatography and high-resolution mass spectrometry. The Global Products Social Molecular Networking database was used to identify the metabolites. We found that few compounds were identified based on the standards and public spectral libraries, suggesting that strain 63 harbors several novel compounds that could protect *C. quadricolor* from infection and predators, as *Streptomyces* sp. SCSIO 001680 has the potential to produce bioactive compounds. Future genomics studies of this strain may reveal novel biosynthetic gene clusters and natural products to help increase the natural product preclinical pipeline.

## Data Availability

Not applicable.

## Supplementary Materials

The following supporting information can be downloaded at: https://www.mdpi.com/article/10.3390/molecules27144542/s1, Figure S1: Molecular network of metabolites produced by *Streptomyces* sp. SCSIO 001680 generated by Cytoscape 3.7.2; Table S1. Spectral information (LC-MS/MS data) for compounds identified through GNPS. For all compounds: Ion source: LC-ESI; MS-Level: MS2; Instrument: qTOF; Ionization mode: Positive; MS Category: Experimental; Table S2. Spectral information for compounds dereplicated on Marinlit. For all compounds: Ion source: LC-ESI; MS-Level: MS2; Instrument: qTOF; Ionization mode: Positive; MS Category: Experimental.
