# Supplementary material for: Metabolomic Profiling and Molecular Networking of Nudibranch-Associated Streptomyces sp. SCSIO 001680
Source: Molecules. 2022 Jul 16;27(14):4542. doi: 10.3390/molecules27144542 (PMC9321954; doi:10.3390/molecules27144542)
Supplement: Supplementary file 1 [file molecules-27-04542-s001.zip › molecules-1815541-supplementary.pdf]

Metabolomic Profiling and Molecular Networking of Nudibranch-Associated *Streptomyces* sp. SCSIO 001680

Samar M. Abdelrahman [1,2,*], Noura S. Dosoky [3], Amro M. Hanora [4] and Nicole. B. Lopanik [1,5,6]

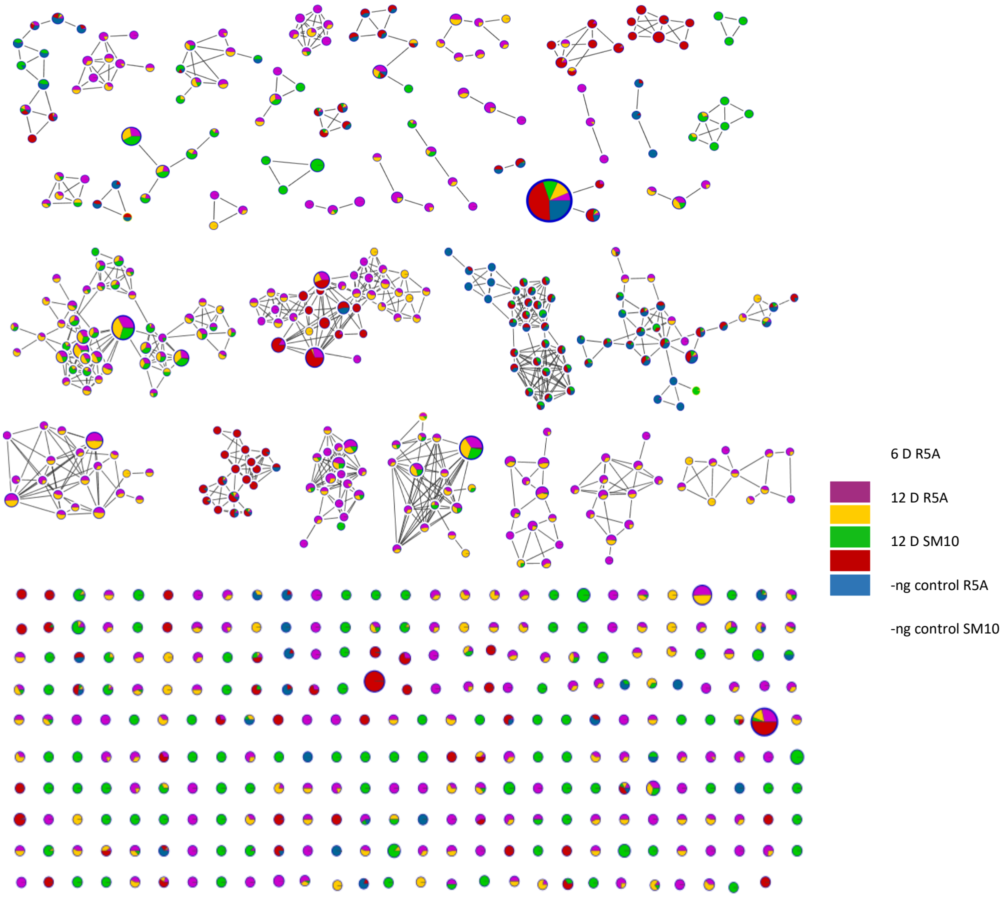

Figure S1. Molecular network of metabolites produced by *Streptomyces* sp. SCSIO 001680 generated by Cytoscape 3.7.2.

Table S1. Spectral information (LC-MS/MS data) for compounds identified through GNPS. For all compounds: Ion source: LC-ESI; MS-Level: MS2; Instrument: qTOF; Ionization mode: Positive; MS Category: Experimental

Compound	MoNA ID	Exact Mass	Precursor Type	Precursor m/z	Top 5 peaks
Marineosin A	CCMSLIB00000001623	409.2837	M+H	410.2909	393.2656<br>361.2434<br>333.2522<br>319.2020<br>291.2099
Desferrioxamine E	CCMSLIB00000001622	600.348	M+Na	623.339	601.3568<br>201.1657<br>183.1604<br>102.1711<br>84.1714
Desferrioxamine D2	CCMSLIB00004698381	586.3326	$[M+Na]^+$	609.322	509.31<br>409.22<br>309.21<br>223.14<br>123.09
Deferoxamine	CCMSLIB00005435927	560.353	M+H	561.3634	561.3634<br>319.2555<br>243.1670<br>201.1656<br>102.1716
Bisucaberin	CCMSLIB00005724304	400.232	M+H	401.25	401.2513<br>301.1620<br>201.1663<br>165.1553<br>102.1715

Table S2. Spectral information for compounds dereplicated on Marinlit. For all compounds: Ion source: LC-ESI; MS-Level: MS2; Instrument: qTOF; Ionization mode: Positive; MS Category: Experimental.

Compound	Energy level	Precursor Type	Precursor m/z	Exact Mass	Top 5 peaks
Halichoblelide	2	$[M+H]^+$	1039.5786	1038.5763	1039.5786<br>1021.5662<br>955.5907<br>977.5794<br>959.5669
Streptodepsipeptide P11B	2	$[M+H]^+$	1083.6068	1082.5999	1083.6068<br>1065.5952<br>1039.6166<br>1021.6066<br>1003.5950
Alternarin A	0	$[M+H]^+$	495.2402	494.2338	495.2402<br>309.1772<br>189.1803<br>126.1707<br>84.1720
Alexandrolide	0	$[M+H]^+$	528.3527	527.3458	528.3527<br>510.3418<br>212.1471<br>196.1563<br>156.1374
Pachastrelloside A	1	$[M+H]^+$	783.4519	782.4452	721.4529<br>407.2175<br>331.2093<br>137.1232
Didemnaketal F	0	$[M+H]^+$	853.4938	852.4871	835.4829<br>725.4085<br>707.3994<br>675.3744<br>319.2104
